# Graphene-Based Materials Immobilized within Chitosan: Applications as Adsorbents for the Removal of Aquatic Pollutants

**DOI:** 10.3390/ma14133655

**Published:** 2021-06-30

**Authors:** Daniele C. da Silva Alves, Bronach Healy, Tian Yu, Carmel B. Breslin

**Affiliations:** 1 Department of Chemistry, Maynooth University, Maynooth, W23 F2H6 Kildare, Ireland; Daniele.CostaDaSilvaAlves.2021@MUMAIL.IE (D.C.d.S.A.); bronach.healy.2017@mumail.ie (B.H.); Tian.Yu.2020@mumail.ie (T.Y.); 2 School of Chemistry and Food, Federal University of Rio Grande, Rio Grande 96.203-900, Brazil

**Keywords:** graphene oxide, chitosan, adsorbent, adsorption, environmental contaminants, magnetic adsorbents, 3D graphene, cyclodextrins, heavy metal ions, dyes

## Abstract

Graphene and its derivatives, especially graphene oxide (GO), are attracting considerable interest in the fabrication of new adsorbents that have the potential to remove various pollutants that have escaped into the aquatic environment. Herein, the development of GO/chitosan (GO/CS) composites as adsorbent materials is described and reviewed. This combination is interesting as the addition of graphene to chitosan enhances its mechanical properties, while the chitosan hydrogel serves as an immobilization matrix for graphene. Following a brief description of both graphene and chitosan as independent adsorbent materials, the emerging GO/CS composites are introduced. The additional materials that have been added to the GO/CS composites, including magnetic iron oxides, chelating agents, cyclodextrins, additional adsorbents and polymeric blends, are then described and discussed. The performance of these materials in the removal of heavy metal ions, dyes and other organic molecules are discussed followed by the introduction of strategies employed in the regeneration of the GO/CS adsorbents. It is clear that, while some challenges exist, including cost, regeneration and selectivity in the adsorption process, the GO/CS composites are emerging as promising adsorbent materials.

## 1. Introduction

The rapid development of industry, agriculture and urbanization, coupled with multiple human activities that can negatively impact the environment, has led to environmental pollution, and especially the contamination of water bodies [1]. Harmful pollutants in water, such as heavy metal ions, organic pollutants and chemical dyes, represent sources of toxicity and create the potential for bio-accumulation and contamination of the aquatic food chain [2]. The continuous developments in novel techniques and routes that are capable of providing clean and safe water have become a significant interest for scientists [3]. Various technologies, such as electrochemical precipitation [4], ion exchange [5], reverse osmosis [6] and photocatalytic degradation [7], have been explored in the removal of pollutants from aqueous solutions. However, these methods have certain limitations. For example, the additional reagents employed in chemical reduction/oxidation may cause secondary pollution [8], while the electrochemical treatment has additional operating costs and the precipitation of sludge is difficult to avoid and requires careful management [9]. Thus, adsorption is considered to be one of the most promising treatment technologies because of its cost-efficiency, ease of operation, simplicity, flexibility and the absence of secondary pollution [10,11].

Graphene, a two dimensional (2D) carbonaceous material with a high specific surface area and very good stability, is emerging as a candidate in the fabrication of adsorbent materials [12]. It has been used as an adsorbent to remove various pollutants from water [13,14] and it also has very good adsorption capacity for gaseous molecules [15,16]. Likewise, graphene oxide (GO) has drawn much attention as it possesses a high surface area, a π-electron system and abundant oxygen-containing functional groups. In addition, studies have shown that the performance of GO as an adsorbent material can be improved by functionalization of GO with a number of reagents [17,18]. Nevertheless, for regeneration and reuse, the collection of GO-based materials from water is an issue, and new simple and efficient removal methods are needed before graphene or GO can be employed in practical applications of water treatment [19]. Moreover, there are strong π–π interactions between GO sheets which result in aggregation, lowering of the surface area, poor dispersion in aqueous media and reduced adsorption efficiency, thus limiting its further use in wastewater treatment [20]. Therefore, new strategies aimed at minimizing aggregation, leaching and recovery of the graphene and/or GO sheets are required, and their functionalization and combination with, or immobilization within, other materials may provide possible solutions. Chitosan is one possible companion material.

Chitosan (CS), a well-known biopolymer, is a promising environmentally-friendly adsorbent due to its biodegradability, non-toxicity and physicochemical properties [21]. It can be used as an immobilization matrix for graphene since it has good receptivity to changes in its structure, while its functional groups, –OH and –NH_2_, can not only act as active adsorption sites, but can also participate in electrostatic interactions and hydrogen bonding with the functional groups on GO, anchoring the GO within the chitosan matrix. On the other hand, this biopolymer has poor thermal stability and mechanical properties, but these properties can be improved and enhanced when chitosan is impregnated with graphene. The combination of these two materials is clearly beneficial with an improvement in the mechanical, thermal and chemical stability of chitosan. In return, the biopolymer acts as a stabilizer for the GO sheets minimizing their aggregation [22].

Given the scientific interest in graphene and GO and the increasing attention that these materials are receiving as adsorbents for the elimination of various contaminants from aquatic environments, a number of review articles have already been published describing the applications of GO as an adsorbent material [23,24,25,26,27]. Moreover, the performances of magnetic GO [28] and 3D graphene-based adsorbents [29,30] have been reviewed recently. Here, we concentrate on the applications of GO/CS composites as adsorbents for the removal of pollutants from aqueous environments. Initially, we focus on the adsorption properties of GO as many of the approaches used to improve its potential as an adsorbent can be applied to the GO/CS system. This is followed by a short introduction to chitosan, its properties and applications as an immobilizing matrix for graphene. Next, we review the GO/CS adsorbents and the additional support materials utilized with GO/CS, providing a comprehensive description of the progress being made in combining these two complementary materials and their performances as adsorbents for the removal of aquatic pollutants.

## 2. Graphene as an Adsorbent Material

In recent years, graphene has attracted considerable attention [31], and it has been employed in numerous applications, ranging from energy storage [32], sensors [33] and electro-Fenton [9] to microwave absorbers [34]. It is also finding applications as an adsorbent material for environmental applications and it is a very welcomed emerging material in this sector as the quality of water continues to decline with increasing amounts of pollutants escaping into the aquatic environment [35,36,37].

### 2.1. Adsorption at Graphene Oxide

Graphene can be synthesized using techniques such as chemical vapour deposition [38] and epitaxial growth [39] to give pristine graphene, but its derivative, graphene oxide (GO), which is decorated with oxygen-containing functional groups, is readily formed using the well-known modified Hummers method [40,41,42]. This process can be used to produce GO on a large scale and involves the oxidation of bulk graphite, a cost effective and abundant material. The resulting GO can then be exfoliated to give GO sheets. Single GO sheets can be generated but the exfoliated GO normally consists of more than one sheet and may exist as a few or multiple sheets. It is this oxidized form of graphene, GO, decorated with hydroxyl, carbonyl, carboxyl, phenol, epoxy, lactone and quinone groups [43], that is attracting considerable attention in the removal of contaminants from water. As an adsorbent material, GO has a number of attractive properties, including a high theoretical specific surface area [44], and the potential for high adsorption capacity. This combined with its very good stability, good thermal and mechanical properties and facile functionalization makes GO an especially interesting material for the removal of contaminants from water [45]. 

The impressive adsorption capacity of GO has been explained in terms of electrostatic, ion exchange, π–π and hydrophobic interactions [17,46,47]. At near neutral pH, GO adopts an overall negative charge, as the acidic groups, such as –COOH, are dissociated. This facilitates the adsorption of positively charged species, such as heavy metal cations, cationic dyes and other cationic molecules through electrostatic interactions [48,49]. Indeed, it has been reported that GO remains negatively charged throughout a wide pH range, typically between 2–11 [50]. However, as the pH increases, the GO becomes more negatively charged, as the equilibrium shifts in favour of the dissociated carboxylate anion, resulting in a higher removal efficiency for cationic dyes. For example, the cationic dye molecule MB (methylene blue) exhibits a maximum adsorption capacity at a pH of 10.0 with GO powders dispersed in the MB-containing solution [50]. Ion exchange has also been proposed as the adsorption mechanism for the removal of heavy metal cations at low pH, where the metal cations are exchanged with the protons in the COOH and OH functional groups [51].

The π–π interactions between aromatic ring structures and the GO sheets can also facilitate the adsorption of pollutants with aromatic ring segments. Nevertheless, the electrostatic interactions tend to be stronger, and this is seen when the aromatic pollutants also contain a cationic group [46]. Hydrophobic effects are seen when the GO polar groups are removed and this can be achieved by reducing the GO sheets to give rGO with a low density of polar functional groups and very good hydrophobicity. In this case, hydrophobic interactions play a role in the adsorption process when the pollutant molecule possesses suitable hydrophobic groups. This is especially relevant in the remediation of oil spills in water, and various graphene-based adsorbents with good hydrophobic properties have been employed to remove oils from water [44,52]. 

Nevertheless, as an adsorbent material, GO has several issues when it is used without any further modifications or not combined with other additives. For example, the GO sheets tend to aggregate and this reduces the adsorption efficiently. Moreover, the number of oxygen-containing groups on GO is relatively low, and while the density of these groups will depend on the degree of oxidation of the GO sheets, there is always an insufficient number of these groups to bind with cationic pollutants. Besides, these oxygen containing functional groups, such as hydroxyl, carboxyl, carbonyl, epoxy and quinone groups, are less effective in binding pollutant molecules compared with nitrogen-based functional groups, such as amines. Therefore, it is no surprise that GO has been functionalized with various nitrogen-containing groups and these have included amino acids [53,54,55], ethylenediamine [56], thiourea [57] and a variety of amino-containing reagents [58,59]. The adsorption capacity of these nitrogen-containing groups is pH dependent, as protonated amine groups, NH_3_^+^, are generated when the pH is decreased. However, at near neutral pH values, these groups become deprotonated, facilitating the binding of heavy metal cations through chelation. 

Selectivity in the adsorption process is especially significant in terms of water treatment, where the water may contain cations, such as Na^+^ and Mg^2+^, and anionic species, such as Cl^–^ and SO_4_^2–^. These have the potential to compete with the adsorption of the targeted contaminants. Different strategies, mainly focussed on molecular recognition, are emerging as possible solutions to the lack of the selectivity associated with GO. One of the more well developed approaches involves the use of cyclodextrins. Recently, there has been an increasing number of publications describing the incorporation of cyclodextrins at GO-based adsorbents [60,61,62]. Cyclodextrins (CDs) are macrocyclic oligosaccharides with a distinctive truncated cone structure that possess a cavity that can include molecules [63,64]. The cavity size differs to give the well-known α, β and γ-CDs. These CDs can incorporate a large variety of guest molecules (host-guest inclusion complexation) making them interesting in drug delivery [65], the development of sensors, [66,67] and as adsorbent materials [68]. In addition, the hydroxyl groups on the CD (7 primary and 14 secondary –OH groups for β-CD) are known to form stable complexes with metal ions. Examples of some β-CD modified GO-based materials and their performances in the adsorption of several contaminants are summarized in Table 1. These CD modified GO-based adsorbents can be formed using a simple self-assembly method, where the CDs, or functionalized CDs, are physically mixed with GO [69]. Self-assembly is favoured as hydrogen bonding occurs between the –OH groups on the CDs and the oxygen-containing groups on GO [66,70]. Alternatively, crosslinking can be used to link the CDs through covalent attachment to the GO [71].

Another emerging option is to use molecular imprinted polymers (MIPs) as the recognition element. MIPs are synthetic polymers tailored to recognise and bind a specific target [78]. This is achieved by polymerization of the monomers in the presence of a template molecule, which is structurally related to the target adsorbent. Once this template is removed from the polymer, binding sites complementary to the target adsorbent are generated to give very impressive selectivity. Besides, the formation of MIPs at GO has the potential to give large surface areas and high adsorption. For example, Cheng et al. [79] formed GO/MIP using GO as the support, bis(2-ethylhexyl) phthalate as the template molecule, methacrylic acid as the functional monomer and ethylene dimethacrylate as the cross-linking agent. The GO/MIP was then employed in the selective extraction of bis(2-ethylhexyl) phthalate. Similarly, Cheng et al. [79] used GO/MIP for the selective adsorption of naphthalene-derived plant growth regulators in apples.

These additional functional groups, CDs and MIP can enhance the removal of several contaminants; however, the GO sheets/nanosheets are nevertheless difficult to remove following the adsorption step. The GO sheets are often used as powders, requiring techniques that are not always suitable in real wastewater treatment, such as centrifugation and filtration, for their removal. In addition, they can leach into the aquatic environment and cause secondary pollution [80]. This leaching can also occur during the adsorption process, making it difficult to control. Even though the toxic effects of GO are poorly understood, there is substantial evidence to show that GO has the ability to penetrate through the cell membranes of aquatic organisms [81,82]. Moreover, GO has the capacity to carry polycyclic aromatic hydrocarbons to aquatic organisms, causing significant toxicity [83]. In order to minimize the leaching of GO, the GO sheets must be immobilized within a support that will facilitate their removal from the aquatic environment, while at the same time maintaining their attractive adsorption potential. 

### 2.2. Recovery of the GO Adsorbent

**Figure 1:**
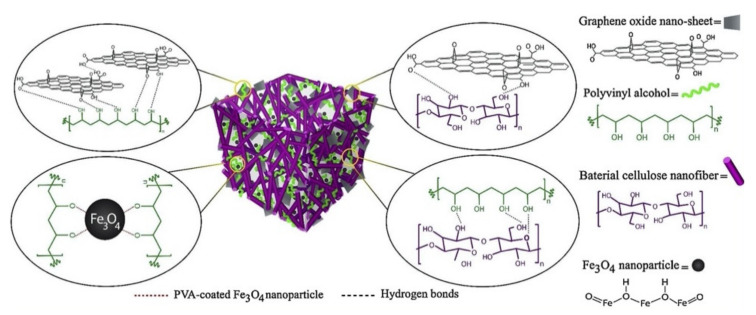
Schematic illustration of a 3D magnetic GO-polymer aerogel. Reproduced with permission from Arabkhani and Asfaram [86], J. Hazard. Mater.; published by Elsevier, 2020.

One approach that is attracting attention is the use of 3D graphene materials [84,85,86]. The development of these 3D hierarchical architectures is challenging as it relies on maintaining the properties of the individual GO nanosheets. These 3D graphene structures include sponges, aerogels and foams, and provided this 3D structure is maintained and does not collapse, these materials are more easily recovered from the solution phase. Moreover, the high surface areas and porous structures give rise to the efficient transport and trapping of the pollutants, while the 3D structures can be further functionalized using covalent and non-covalent methodologies [87,88]. The various methods used in the formation of porous 3D graphene structures can be found in a recent review by Lin et al. [30], highlighting the increasing interest in 3D graphene-based materials. In Figure 1, a schematic illustration of a 3D GO-based magnetic polymeric aerogel is shown, where the 3D network was formed using freeze-drying and contains magnetic Fe_3_O_4_ nanoparticles, polyvinyl alcohol (PVA), cellulose and GO sheets.

Another strategy involves the use of magnetic graphene-based adsorbents and these materials are attracting considerable interest in the adsorption of pollutants from water [89,90,91]. The magnetic properties are normally introduced using Fe_3_O_4_, a ferromagnetic black iron oxide, which possesses very good compatibility, low toxicity, very good magnetic properties and which can be generated as rods, wires, spheres and nanoparticles [92]. The addition of the magnetic particles not only facilitates the separation of the adsorbent from the aquatic environment through a simple magnetic process [93], but also can form between the GO sheets and reduce the inevitable aggregation of the sheets.

### 2.3. Immobilization of GO within Biopolymers

Clearly, GO can be modified with various additives aimed at improving adsorption, the selectivity of the adsorption process and the recovery and removal of the adsorbent following the adsorption step. However, the immobilization of GO using support materials that can provide a stable matrix to securely anchor the GO, while maintaining its impressive high surface area, mechanical strength and adsorption capacity is still a challenge. Biopolymeric/GO-based adsorbents are now attracting increasing attention as they are environmentally acceptable and can be easily synthesized. Besides, the biopolymer backbone possesses interesting functional groups that have the ability to bind environmental contaminants. Several biopolymers have been combined with GO and employed in the adsorption of environmental contaminants, including cellulose [94], alginate [95], gum [96] and lignin [97]. However, within the family of biopolymers, chitosan is the leading candidate and is attracting increasing interest as a support matrix for GO, rGO and graphene. Using Scopus and the key words ‘graphene’ and ‘chitosan’, a total of 2226 publications were found, with 75 publications in 2012 and 413 in 2020. These chitosan/GO composites are gaining attention in a number of applications and especially as adsorbent materials. They can be used as powders and dispersed in the solution phase, but also employed as solid adsorbents, enabling their removal and recovery from the treated water, while limiting the aggregation of the GO sheets, arising from intermolecular forces. Their impressive adsorption potential is described following a short introduction to chitosan and its properties related to its adsorption potential.

## 3. Chitosan as an Immobilization Matrix 

**Figure 2:**
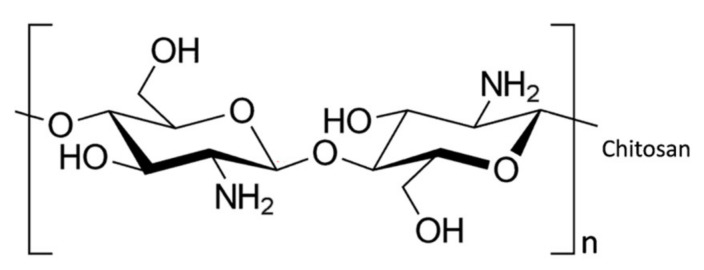
Chemical structure of chitosan.

Chitosan, a readily available eco-friendly and non-toxic polysaccharide, is fabricated from chitin, and consists of β–(1–4)–D–glucosamine. It has very good chemical stability combined with chelating properties [98]. Indeed, chitosan has been employed as an effective adsorbent for the removal of dyes [99], phenol [100], heavy metal ions [101], antibiotics [102] and pesticides [103] from water. It is an effective adsorbent as it has a high surface area and possesses a large density of hydroxyl (–OH) and primary amine (–NH_2_) groups, as illustrated in Figure 2. These functional groups can serve as active adsorption sites which facilitate the adsorption and removal of both positively and negatively charged molecules through electrostatic interactions [104,105]. In particular, amine groups are strongly attracted to metal ions through ion–dipole interactions [101], while the protonation of these amine groups (–NH_3_^+^) facilitate electrostatic attraction of anionic compounds, including halides [106] and anionic dyes [107]. 

Chitosan is an interesting immobilization matrix for graphene as it can be modified chemically through cross-linking, complexation and grafting, while different functional groups can also be introduced [105,108]. Furthermore, parameters such as the molecular weight (MW), deacetylation degree (DD), solubility, crystallinity, particle size and surface area can all be optimized to enhance the adsorption capacity [109]. The MW of chitosan depends on the deacetylation process, the source and the preparation procedures employed in its formation [110]. It can affect many of the physicochemical properties of chitosan, including its crystallinity, solubility, viscosity, tensile strength and elasticity, and impact on the applications of chitosan as an adsorbent material [111,112]. The DD, which is related to the acetyl content in chitosan, can be altered by varying the alkaline treatment step in the chitin deacetylation process [113]. An increase in DD, usually achieved through a repeated or prolonged alkaline treatment, gives rise to an increase in the density of free amino groups. Consequently, the polycationic nature is increased. This higher charge density along the chitosan chain alters the chain flexibility [114]. Indeed, it has been demonstrated that chitosan chains with higher DD have a more regular packing of the polymer chains, which promotes crystallinity in chitosan [115]. Although the stiffness and tensile strength of chitosan are improved on increasing the crystallinity, a reduction in elongation and an increase in the brittleness can also occur. Since the cationic properties of chitosan are connected with DD, it has been shown in several reports that DD affects the adsorption properties of chitosan [116]. For example, Gonçalves et al. [117], in studying the adsorption of dyes at chitosan powders with different DD levels, observed an increase in the adsorption capacity of the dyes on increasing the DD from 75% to 95%. Likewise, Piccin et al. [118] observed a higher adsorption capacity as the DD was increased from 42 to 84%. Furthermore, it has been shown that chitosan composites with higher DD are more stable and reusable, even after 15 adsorption-regeneration steps [113]. Nevertheless, the highly hydrophilic character associated with a high DD may have some limitations [119]. Indeed, Iamsamai et al. [120] showed that better dispersion of multiwalled carbon nanotubes (MWCNTs) was achieved with lower DD levels (61% DD). Therefore, DD can play a key role when chitosan is combined with other materials and employed as an adsorbent [121].

The solubility of chitosan depends on a number of factors, including the density and distribution of amino and N-acetyl groups on the polymeric chitosan chains and the ionic strength of the solution phase [122]. Hence, solubility is linked with both the DD and MW. Under acidic conditions, the amine groups become protonated and the chitosan becomes more soluble. As more amino groups become protonated, achieved with higher DD, stronger electrostatic repulsion occurs between the neighbouring chains and this results in dissolution of the polymer [109]. On the other hand, as the pH of the solution is increased to a value in the vicinity of 6.0, precipitation of the solubilized chitosan occurs as the amine groups become less protonated [123]. On increasing the MW, higher levels of inter- and intra-molecular hydrogen bonding occurs within the chains, and this results in chain entanglement and a concomitant reduction in solubility [124]. Indeed, it has been shown that the solubility of chitosan depends on the pH and ionic strength [125], temperature, time of deacetylation, alkali concentration, previous treatments applied to the isolation of chitin and particle size [126]. Therefore, the solubility is important as it imparts the chitosan with excellent gel-forming properties and these are important in the formation of the GO/CS hydrogel composites.

The porosity and surface area of chitosan can also play a central role in the adsorption process and can have a significant effect on the adsorption capacity [127]. It is well known that the particle size of chitosan is an important characteristic that is related to the porosity, pore size and pore volume [128] and these are fundamental for adsorption applications [129]. For example, a low uptake of pollutants was observed with larger particle sizes and this was explained in terms of a lower surface area [130], while an increase in the surface area results in the formation of new active sites, which allows more binding of contaminants, and consequently an increase in the overall performance of the adsorbent is seen [131]. Nevertheless, it is challenging to obtain a highly porous chitosan material, with good mechanical stability combined with the possibility of regeneration and reuse [132]. 

The relatively poor mechanical stability of chitosan hydrogels is limiting its applications and the addition of reinforcing fillers is a possible strategy to enhance its mechanical properties [133]. GO has been used to form GO/CS composites. This is an interesting combination as the GO can self-assemble with the chitosan chains especially in acidic solutions where the amine groups are protonated. This provides good stabilization and this combination is also very well suited to freeze-drying [134]. In addition, GO can improve the adsorption capacity due to its inherent ability to adsorb certain classes of water pollutants [135]. This GO/CS combination is interesting as the GO can enhance the physiochemical properties of chitosan, while the chitosan can immobilize the GO sheets, minimizing aggregation and minimizing the leaching of GO. In the following section, these GO/CS composites are described, with a focus on their synthesis, preparation and modification methods together with some of their properties and their ability to form adsorbents for the removal of water contaminants.

## 4. Graphene/Chitosan Adsorbents

As detailed in Section 3, chitosan has a number of unique properties that make it an interesting candidate as an adsorbent material, and also as an immobilization matrix for graphene, GO, rGO and 3D porous graphene monoliths [13,136]. Impregnation of graphene into chitosan is a good way to achieve both mutual stabilization and an enhancement in the adsorption capacity [137]. 

### 4.1. Formation of Graphene/Chitosan Adsorbents

**Figure 3:**
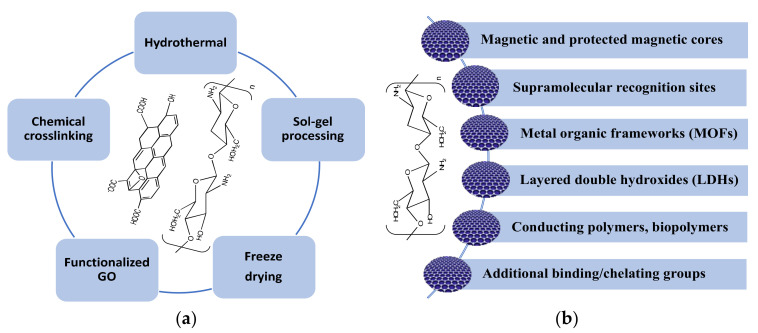
Summary of the (**a**) main processes used to fabricate GO/CS and (**b**) other additives combined with GO/CS.

**Figure 4:**
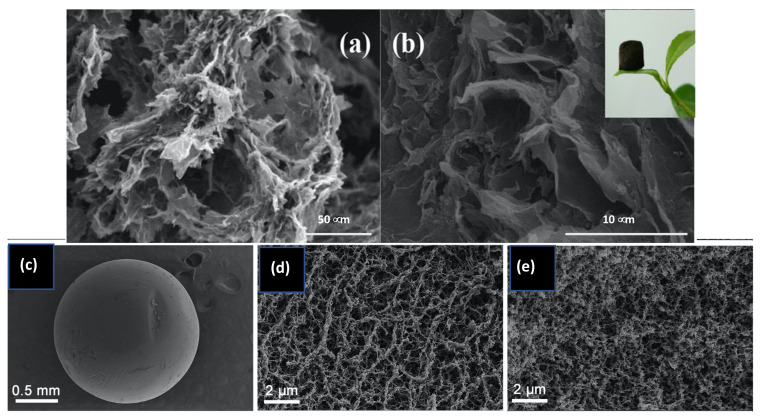
SEM micrographs of GO/CS/LS at magnifications of (**a**): ×600, (**b**): ×5000 and supported on a leaf. Reproduced with permission from Yan et al. [143], Int. J. Biol. Macromol.; published by Elsevier 2019. GO/CS (**c**) beads/spheres, (**d**) surface and (**e**) cross-sectional structures. Reproduced with permission from Wu et al. [140], Mater. Sci Eng. C; published by Elsevier 2020.

Graphene and chitosan composites can be formed using a number of strategies, and the main elements involved in these processes are summarized in Figure 3a. In many cases, these different elements are combined to give the final GO/CS composite. Generally, the first step is dissolution of chitosan in an aqueous solution of acetic acid (1–3% *v/v*), which is one of the most frequently employed solvents for the solubilization of chitosan, to give a yellowish coloured homogeneous solution [138]. Then, GO, or functionalized GO, is added with sonication to form a homogeneous mixture [139]. This mixture can be used to give GO/CS beads [140] or further processed using vacuum-assisted self-assembled filtration (VASA) [141], solvothermal and hydrothermal reactions [142], freeze drying [134] or combinations of these. Some of the advantages and disadvantages of these synthetic processes are summarised in Table 2. Micrographs obtained using scanning electron microscopy (SEM) are presented in Figure 4 and these highlight the surface morphologies of the synthesised GO/CS-based adsorbents. The micrographs presented in Figure 4a,b represent the morphology of GO/CS combined with lignosulfonate (LS) and illustrate the interconnected three dimensional porous network of the fabricated GO/CS/LS. As shown in the inset of Figure 4b, the porous GO/CS/LS is sufficiently light to be supported on a leaf. In Figure 4c–e, GO/CS beads are shown, displaying a spherical shape with little or no defects, while higher magnifications of the surface and cross sections show the typical interconnected network. 

**Figure 5:**
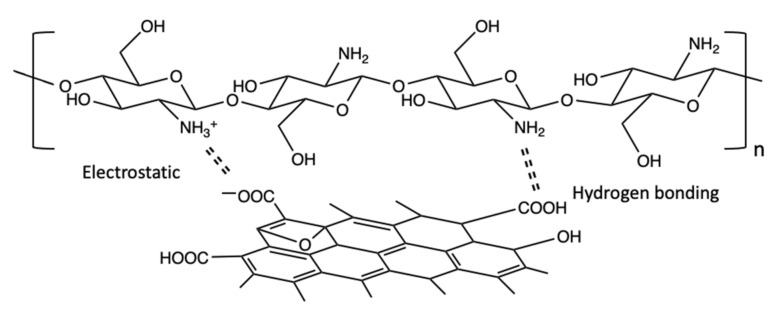
Illustration of interactions between GO and chitosan.

The formation of these GO/CS hydrogel composites is aided by electrostatic interactions, hydrogen bonding and covalent interactions between chitosan and GO [135]. At near neutral pH, the amino groups of chitosan are protonated and these attract the negatively charged groups, such as –COO^–^, on GO, to give a stable hydrogel composite [144]. These electrostatic interactions combined with hydrogen bonding facilitate the formation of the GO/CS hydrogel which provides stable composites with excellent mechanical and thermal properties [145]. Furthermore, the introduction of chitosan to GO nanosheets provides an increase in the surface area, pore size and total pore volume, which facilitate efficient adsorption onto the GO/CS hydrogel surface [146]. Thus, the effective intermolecular interactions between GO and chitosan play an important role in the specific structural formation of the hydrogel nanocomposites and these are illustrated in Figure 5. 

Crosslinking agents have also been added to further enhance the mechanical properties of GO/CS. Reagents such as trisodium citrate, sodium tripolyphosphate [147], glutaraldehyde [104,148], genipin [149,150], borax [151] and N-(3-dimethylaminopropyl)-N-ethylcarbodiimide hydrochloride [152] have been successfully employed. Some of the more commonly used crosslinking reagents are summarised in Table 3, where it is seen that they are normally employed at low concentrations, and both temperature and time can be varied to achieve the crosslinking step. The GO can also be functionalized to facilitate cross-linking with chitosan and one of the more well-known reactions employed is amidation, where the functional groups on GO are activated to give acetyl chloride (–COCl) functionalized GO, which can then be connected with a nitrogen containing group through the formation of –CONH– linkages [152].

These cross-linking agents can play different roles and influence the number of functional groups available to bind with pollutants. For example, borax undergoes hydrolysis to yield the tetrahydroxyborate ions, B(OH)_4_^−^, which react with the hydroxyl groups in chitosan and GO to produce orthoborate chemical bonds. On the other hand, N-(3-dimethylaminopropyl)-N-ethylcarbodiimide hydrochloride, which is water soluble and biocompatible, can be used as a synthetic grafting agent [152], coupling the carboxyl (GO) and amino groups (CS) to give amide bonds. Moreover, the timing of this step can influence the performance of the GO/CS adsorbent. For example, Salzano de Luna et al. [153] demonstrated that freeze dried GO/CS composites were more effective in the adsorption of dyes when the cross-linking was carried out following the freeze drying step. This was attributed to a higher degree of pore interconnectivity in the GO/CS composites crosslinked after freeze-drying. However, the compressive modulus was reduced. These studies highlight the significant contribution of cross-linking, not only in terms of mechanical properties, but also in terms of pore interconnectivity and the provision of chelating binding sites for the uptake and removal of pollutants.

**Table 3:** Summary of some crosslinking agents employed in the fabrication of GO/CS adsorbents.

Crosslinking Agent	Crosslinking Conditions	Advantages/ Disadvantage	Ref.
Gluteraldehyde	2% solution (wt %), 1 h at 60 °C	Inexpensive but exhibits toxicity	[148]
Gluteraldehyde	1% solution (wt %), 6 h at 25 °C		[141]
Gluteraldehyde	2% solution (wt %), 8 h at 30 °C		[154]
Glycidoxypropyltri-methoxysilane (KH-560)	0.22 g KH-560 with 0.12 g GO, 1.5 g CS at 50 °C	Commonly used coupling agent, some toxicity	[155]
Genipin	1% solution (wt %) added dropwise, 1 h at 25 °C	Negligible toxicity	[150]
Borax	10% solution (wt%) 1 h at 25 °C	Toxic	[151]

As illustrated in Figure 3b, additional components are added to further enhance the performance of the GO/CS-based adsorbents. These vary from magnetic particles to blending with other polymers. In most cases, GO is employed; however, the more conducting rGO has also been used and decorated with uncapped metal nanoparticles and then combined with chitosan [156]. As the metal nanoparticles are formed through reduction of the appropriate salt in solution, the rGO is oxidized to GO to give a simple methodology for the decoration of GO with uncapped nanoparticles. Nevertheless, it is generally accepted that the GO/CS-based adsorbents have a higher adsorption capacity due to a combination of π–π stacking, electrostatic interactions and hydrogen bonding with the water contaminants [12,50]. Indeed, Guo et al. [18] found that while both GO/CS and rGO/CS could adsorb dyes, the GO/CS appeared as the more efficient adsorbent. 

### 4.2. Magnetic Chitosan/GO

Magnetic materials have emerged as exciting new materials in several applications [157] and it is no surprise that they have been employed to give magnetic GO/CS (MGO/CS) adsorbents [158,159]. As previously mentioned in Section 2.2, magnetism can be employed to achieve removal of the adsorbent from the aquatic environment [93]. Depending on the synthetic conditions utilized to fabricate the GO/CS adsorbents, it may be difficult to completely remove these adsorbents using techniques such as sedimentation or filtration. The introduction of magnetism provides a simple but effective solution and this has become the focus of a number of recent investigations.

**Figure 6:**
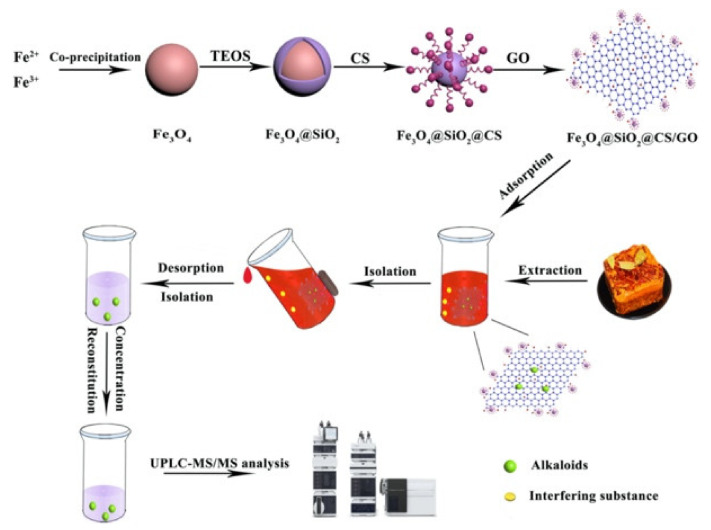
Schematic for the procedure for synthesis of Fe_3_O_4_/SiO_2_/CS/GO and its application in the removal of alkaloids. Reproduced with permission from Tang et al. [168], Int. J. Biol. Macromol.; published by Elsevier 2020.

The MGO/CS composites can be formed using ex-situ methods [160], where the Fe_3_O_4_ nanoparticles are chemically synthesized and then combined with the GO/CS hydrogel [161]. Alternatively, in-situ methods [162,163] can be used. For example, Singh et al. [159] used the amide functional groups (formed between the epoxy (GO) and amine (CS) groups) for the in-situ reduction of iron ions to iron oxides. The synthesized Fe_3_O_4_ nanoparticles have been observed to immobilize on the GO sheets [158,164], but agglomeration is normally seen [92,165,166]. However, Jiang et al. [167] have shown that the severe aggregation, arising from the magnetic properties of the Fe_3_O_4_ particles, can be minimized when the particles are coated with silica. This silica layer not only inhibits aggregation, but also protects the magnetic cores and these silica coated Fe_3_O_4_ particles have been combined with GO/CS and employed in the removal of alkaloids [168], as illustrated in Figure 6. The MGO/CS can be formed as beads [169,170] and as various powders and nanocomposites [171,172]. In addition to providing magnetic separation the added Fe_3_O_4_ nanoparticles, provided they are well dispersed and not agglomerated, can give rise to an improvement in the surface area of the MGO/CS adsorbents [173]. Indeed, these MGO/CS adsorbents have been described as mesoporous materials with surface areas ranging from 37.28 m^2^ g^−1^ [174], 74.345 m^2^ g^−1^ [175], 388.3 m^2^ g^−1^ [176], 392.5 m^2^ g^−1^ [173] to 402.1 m^2^ g^−1^ [177] and pore volumes varying from 0.084 cm^3^ g^−1^ [174], 0.3852 cm^3^ g^−1^ [173] to 0.4152 cm^3^ g^−1^ [177].

The performance of various MGO/CS adsorbents is illustrated in Table 3, where the adsorption capacity, q_m_, is expressed in terms of the ratio of the mass of adsorbate removed to the mass of adsorbent used. These experimental adsorption data are normally fitted to two main adsorption models, the Langmuir [178,179] and Freundlich [180] isotherms, with the Langmuir isotherm being more frequently utilized. In the Langmuir isotherm, all the adsorption sites are considered equivalent with no interactions between adjacent sites. This gives rise to monolayer adsorption. On the other hand, the Freundlich isotherm is based on multilayer adsorption. Although the GO/CS-based adsorbents have a number of different and distinct adsorption sites, the experimental data fit very well with the monolayer adsorption process inherent in the Langmuir model. 

As illustrated in Table 4, the MGO/CS adsorbents are often further modified with cyclodextrins. For example, Li et al. [181] have described the removal of Cr(VI) under acidic conditions to the attraction of the negatively charged chromate anions to the protonated chitosan, reduction of Cr(VI) to Cr(III) at the GO sheets, and the binding of anionic Cr(VI) and cationic Cr(III) at the cyclodextrins. The hydrophobic and inclusion complexation characteristics of the cyclodextrin may also be relevant in the removal of dye molecules and these properties have been exploited to give good removal of methylene blue [177] and hydroquinone [182] at the CD modified MGO/CS. However, it is difficult to make a direct comparison between the adsorption capacity of the adsorbent materials presented in Table 3. Not only are the properties of chitosan, which is very dependent on its production and isolation from chitin, not identical, but the conditions used in the adsorption experiments are different. Interestingly, on comparing the adsorption capacity of methylene blue, which is frequently used as a model pollutant, it is clearly evident that there is considerable variations in its adsorption, with values ranging from 43.34 to 2478 mg g^−1^. These results highlight the importance of well-dispersed and interconnected GO sheets throughout the chitosan to give a porous matrix, while the characteristics of the chitosan employed (for example DD and MW) and the nature of functionalized groups are also very important. Indeed, the highest adsorption capacity is evident with methacrylic acid-functionalized chitosan, which is cross-linked with N,N-methylenebis(acrylamide), to give a solid matrix that facilitates the stable dispersion of both GO sheets and Fe_3_O_4_ nanoparticles. 

Surface ion imprinted MGO/CS adsorbents have also been fabricated with a view to providing enhanced selective uptake and removal of certain heavy metal ions. Wang et al. [194] used this strategy to synthesize Pb-MGO/CS for the selective removal of Pb(II). While the MGO/CS showed no selectivity for the adsorption of Pb(II), the imprinted adsorbent exhibited specific recognition for Pb(II) in a mixed metal ion solution. This was attributed to the cavities created on removal of the Pb template, with the appropriate size, shape and coordination geometry to capture Pb(II). Ion imprinting has also been employed with the MGO/CS adsorbents in the selective removal of Cu(II) [195].

### 4.3. Chitosan/GO with 3D Architectures

As detailed in Section 2.2, 3D GO composites possess a unique 3D porous structure decorated with surface functional groups that have the ability to bind and remove pollutants from water. The 3D network consists of interconnected, curved, wrinkled and distorted graphene sheets to provide a porous 3D structure with high specific surface area [196]. Depending on the shape of the material, sponges, foams, or porous graphene films can be obtained [197]. Although these 3D structures are more easily recovered from the solution phase following the adsorption step compared to GO powders, they can exhibit rather poor stability in water. Typically additional supports are added, but these must be selected and chosen so that the distorted GO sheets are available and free to act as adsorption sites. In this regard, chitosan is particularly suitable as it can be processed by freeze-drying, to give aerogels [21].

These 3D GO/CS porous networks are normally formed by mixing/sonicating all the components, including GO or rGO, chitosan and any crosslinking agents or other additives, followed by some thermal processing and a final freeze-drying step [198,199,200]. Alternatively, a 3D scaffold can be employed as a template. For example, a polylactic acid (PLA) 3D scaffold was printed and then immersed in a GO/CS mixture, followed by freeze-drying to give a 3D sponge [201], while aerogel microspheres were prepared by combining electrospraying and freeze-casting [202]. Likewise, Kovtun et al. [203] used aerogels comprising 3D chitosan-gelatin. These were then modified with GO by either embedding the GO sheets within the aerogel or by coating the surface of the aerogel with GO sheets. These two methods were compared in the adsorption of ofloxacin and ciprofloxacin (fluoroquinolonic antibiotics) and Pb(II). It was concluded that the adsorption of Pb(II) was fast at the GO surface coated aerogels, where the adsorption sites were easily and rapidly accessed. On the other hand, diffusion of Pb(II) to the embedded GO required a longer contact time for adsorption. 

These 3D GO/CS adsorbents have shown relatively good stability and reuse in the removal of reactive black 5 dye [134]. Likewise, good recyclability and stability was achieved with GO/CS sponges, with a regeneration efficiency in excess of 80% over five cycles in the removal of heavy metal ions [204]. Other contaminants that have been removed successfully using 3D GO/CS composites include dyes [146,153], Cu(II) [205], Cr(VI) [206], tetracycline [207] and 4-nonylphenol [208]. The incorporation of β-cyclodextrins into this 3D network can be employed to further enhance adsorption. For example, 3D-GO/CS/β-CD was employed as an effective adsorbent for MB yielding an ultrahigh adsorption capacity of 1134 mg g^−1^ [198]. Other additives include montmorillonite, a well-known adsorbent [199], kaolin, a filler employed to increase the mechanical strength of the adsorbent [209], and magnetic nanoparticles [200]. One of the more significant advantages of using these 3D GO/CS networks is the reuse and recyclability of the adsorbent without the need for a complex and time-consuming filtration process [198]. Moreover, they exhibit large surface areas, while the interconnected pores enable diffusion of the adsorbate throughout the 3D network leading to high adsorption capacity.

### 4.4. GO/Chitosan and Additional Chelating Agents

The GO/CS adsorbents have a number of binding sites for adsorption, but some of these are consumed during the crosslinking steps and this can involve the removal of essential −OH and −NH_3_^+^ groups, as illustrated earlier. However, the density of appropriate functional groups can be enhanced through the addition of chelating agents and this strategy has been employed with the aim of increasing the adsorption capacity. Some of the chelating agents employed with the GO/CS adsorbents and their performances in the adsorption of various contaminants are provided and summarized in Table 5. EDTA, an amino carboxylic acid with four carboxylic acid groups, is one of the more popular chelating agents as it can scavenge or chelate various cationic species. 

**Figure 7:**
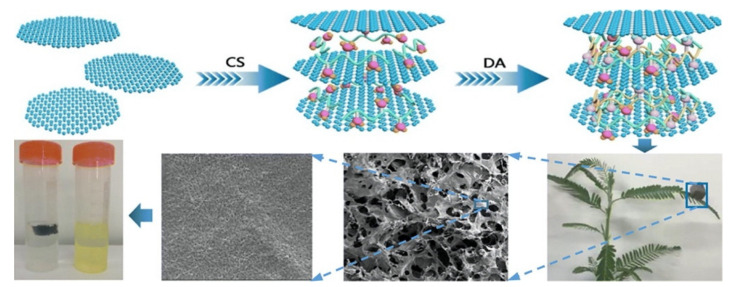
Schematic illustration of the preparation of graphene oxide/chitosan/polydopamine (GO/CS/PDA). Reproduced with permission from Li et al. [211], Int. J. Biol. Macromol; published by Elsevier, 2020.

Another emerging chelating additive is polydopamine (PDA). This is easily generated from the oxidation and self-polymerization of dopamine in slightly alkaline solutions [210]. Polydopamine provides both amine and catechol groups and can also participate in hydrogen bonding and π−π stacking interactions, making it a very good chelating agent that can be easily combined with GO/CS. A schematic illustration of these interactions and the assembly of GO/CS/DPA is shown in Figure 7, where the added PDA is sandwiched between the GO sheets, serving to minimise aggregation [211]. Moreover, the PDA with abundant catechol and amino groups can serve as an active surface for functionalization. For example, Cao et al. [212] used a Michael addition reaction with the thiol group of 1H,1H,2H,2H-perfluorodecanethiol (PFDT) to create perfluorinated rGO/CS-PDA with superhydrophobic properties. This functionalized adsorbent was then utilized as a cost-effective and environmentally-acceptable approach for the separation of oil/water mixtures. 

Lignosulfonate (LS) is a further example of a chelating agent with abundant sulfonic (−SO_3_^−^) and hydroxyl (−OH) groups and with a strong affinity for the binding of metal ions and charged molecules. This polyelectrolyte has been combined with GO/CS and employed in the adsorption of the cationic methylene blue [186]. As shown in Table 5, these additional chelating agents, PDA, EDTA and LS, are effective in the adsorption of a variety of heavy metal ions. In particular, very good removal of Pb(II) is evident, while the presence of these additional chelating agents also facilitate the removal of tri-valent cations.

**Table 5:** Chelating agents combined with GO/CS-based adsorbents, where the solid adsorbents are added to the pollutant-containing solution.

Chelating Agent	Adsorbent/Adsorption Experiment	Adsorbate	Adsorption q_m_ (mg g^−1^)	Ref.
Ethylenediaminetetraacetic acid (EDTA)	MGO/CS/EDTA Batch, 25 °C, 180 rpm, 0.33 g/L adsorbate, V = 30 mL	Pb(II) Cu(II) As(III)	206.5 207.3 42.7	[179]
EDTA	GO/CS/EDTA Batch, 25 °C, 160 rpm, 20 mg adsorbent, V = 50 mL	Cr(VI)	86.2	[180]
EDTA	MGO/CS/EDTA Batch, 33 °C, 0.14 g/L adsorbent, 114 mg/L adsorbate	Rhodamine B	1085.3	[213]
EDTA	MGO/CS/EDTA Batch, 20 mg adsorbent, V = 15 mL	Pb(II)	970	[214]
EDTA	MGO/CS/EDTA Batch, 49.2 °C, 40 Hz sonication, 9.5 mg adsorbent, V = 50 mL	Pb(II)	666.6	[161]
Polydopamine (PDA)	GO/CS/Polyvinyl alcohol (PVA)/PDA Batch, 40 °C, 150 rpm, 50 mg adsorbate	Cu(II) Pb(II) Cd(II)	210.9 236.2 214.9	[215]
PDA	GO/CS/PDA aerogel Batch, 25 °C, 120 rpm, 0.3 g/L adsorbent	U(VI)	415.9	[216]
PDA	GO/CS/PDA Batch, 25 °C, 150 rpm, 15 mg adsorbent, V = 20 mL	Cr(VI)	312.0	[211]
PDA	MWCNT/PDA/GO/CS Batch, 25 °C, 10 mg adsorbent, V = 10 mL	Gd(I)	150.8	[217]
PDA	GO/CS/PDA Batch, 30 °C, adsorbate 500 mg/L, V = 100 mL	Cu(II) Ni(II) Pb(II)	170.3 186.8 312.8	[218]
Lignosulfonate (LS)	MGO/LS/CS Batch, 30 °C, 160 rpm, 10 mg adsorbent, V = 20 mL	Methylene blue	50	[186]
LS	GO/LS/CS Batch, 30 °C, 130 rpm, 0.2 g/L adsorbent, V = 25 mL	Methylene blue	1023.9	[143]

### 4.5. GO/Chitosan Combined with Other Adsorbent Materials

The GO/CS hydrogel provides an attractive matrix for encapsulating other adsorbent materials and especially powdered materials that are difficult to separate and recover after the adsorption process, limiting their environmental applications. Consequently, there is much interest in combining other adsorbents with the GO/CS hydrogels. For example, metal–organic frameworks (MOFs), which consist of metal ions and polyfunctional organic ligands and have good adsorption potential, have been successfully integrated within GO/CS for the elimination of Cr(VI) [219]. Moreover, they have been employed as both an adsorbent and photocatalyst in the removal of methylene blue, reaching an adsorption capacity of approximately 357.15 mg g^−1^ [220]. The intercalated MOF can also provide the GO channels with molecular-sieving properties. Using this strategy, Chang et al. [221] prepared a GO/CS/MOF membrane for the purification of water. The GO/CS/MOF membrane exhibited very good water flux (14.62 L m^−2^ h^−1^ bar^−1^), with high rejection (>99% for dyes) and good antifouling characteristics.

**Figure 8:**
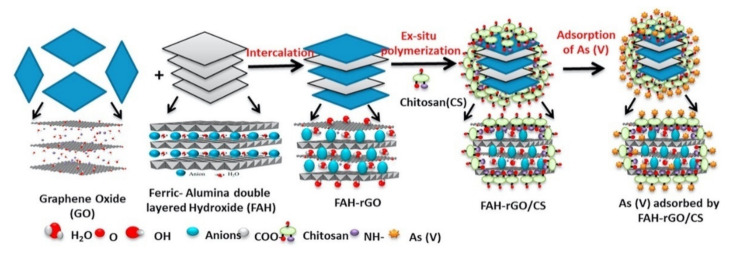
Schematic of the formation of GO/CS/LDH(Fe-Al) and illustration of the adsorption of As(V). Reproduced with permission from Priya et al. [223], Mater. Chem. Phys.; published by Elsevier, 2020.

Hydroxyapatite (Hap) is another promising adsorbent with good bioactive, non-toxic and biocompatible properties. Its adsorption capacity can be enhanced by combining it with other suitable adsorbent materials and GO/CS with its π–π stacking, hydrogen bonding and electrostatic interactions is a suitable host material. GO/CS/Hap has shown good adsorption of dye molecules with adsorption capacities of 43.06, 41.32 and 40.03 mg g^−1^ for the removal of Congo Red, Acid Red 1 and Reactive Red 2 from water, respectively [129]. Montmorillonite is an alternative cost effective, sheet-like adsorbent material that has rather poor adsorbent capacity as an individual material, but it has been shown to enhance the stability of rGO/CS [199]. This porous hydrogel was formed without any cross-linking of chitosan and served as an efficient adsorbent for the uptake of Cr(VI) (87.03 mg g^−1^) [199]. Another candidate is layered double hydroxides (LDHs). These 2D materials consist of layers of divalent and trivalent cations, with intercalating anions. These layered materials have been employed in the removal of heavy metal ions [222]. More recently, they have been integrated with GO/CS hydrogels to give improved adsorption performances [148,223]. In Figure 8, a schematic describing the formation of GO/CS combined with Fe-Al double layered hydroxide is shown illustrating its application in the removal of As(V) [223]. 

Antifouling reagents, such as ZnO and silver ions, can also be easily integrated within the GO/CS hydrogels and used to increase antibacterial activity. For example, GO/CS/ZnO hybrid composites have shown impressive antibacterial activity against *E. coli* and *S. aureus* and very good adsorption of methylene blue [224], while GO/CS/MOF modified with silver ions, with good antibiofouling characteristics, was employed for the adsorption of uranium [225].

### 4.6. GO/CS and Polymer Blending and Hybrids

Although chitosan has several attractive properties as an immobilization matrix for GO, it can suffer from relatively poor hydrolytic stability. In an effort to overcome these technological challenges, chitosan copolymers and the blending of chitosan with other synthetic polymers has received considerable attention. A number of synthetic polymers, with well-defined structures, and various biopolymers, with less defined structures, have been combined with chitosan and GO. The polymers employed include poly(vinyl alcohol) (PVA) [226], polyacrylic acid (PAA) [227], polylactic acid (PLA) [201], cellulose (C) [228], carboxymethyl cellulose (CMC) [229] and alginate [169]. The performance of these chitosan blended polymer systems as a matrix for GO is summarized in Table 6, where it is readily evident that these systems have good adsorption capacity. 

Conducting polymers, such as polypyrrole [136,234,235], have also been added to the GO/CS hydrogel. This can be easily achieved through the in-situ polymerization or electropolymerization of the corresponding monomer within the GO/CS hydrogel. The addition of conducting polymers, such as polypyrrole, which have very good stability and a porous and high surface area, has the potential to enhance the adsorption capacity as the conducting polymers can capture charged contaminants as dopants, participate in hydrogen bonding and be involved in π–π interactions. Magnetic nanoparticles can also be easily deposited at the conducting polypyrrole to give hybrids which can be removed following adsorption using magnetic separation [236]. 

## 5. Adsorption and Regeneration Processes

The ideal adsorbent should possess a high adsorption capacity, but it should also be possible to desorb the adsorbate and regenerate the adsorbent. These processes are now described and discussed.

### 5.1. Adsorption Capacity 

The adsorption process depends on a number of experimental parameters, such as the initial concentration of the adsorbate, the pH and ionic strength of the solution, temperature and adsorption contact time. The pH of the solution is one of the more significant parameters as it influences both the surface properties of the adsorbent and ionization state of the adsorbates, and therefore affects significantly the electrostatic interactions. Typically, the GO/CS adsorbents have a pH of zero charge (pH_pzc_) ranging between about 2.2 [143] and 6.0 [237]. Accordingly, the GO/CS adopts an overall positive charge under acidic conditions and a more negative charge at higher pH values. Generally, the adsorption capacity for divalent metal ions, such as Cu(II), increases from a pH of about 2.0 to 7.0, but then decreases rapidly at higher pH values [187,238]. This can be explained in terms of the formation of protonated chitosan under acidic conditions, where the resulting NH_3_^+^ groups repel cations. However, as the pH increases and the GO/CS adopts an overall negative charge, with the generation of –COO^–^ and –NH_2_ groups, electrostatic interactions are favoured and the cations can be eliminated from the solution phase. As the pH is further increased, insoluble metal hydroxides are formed leading to precipitation in the solution phase. In terms of the removal of Cr(VI), the anionic dichromate (Cr_2_O_7_^2–^) and chromate (HCrO_4_^–^) species are generated at pH values in the vicinity of 2.0 to 4.0, giving higher adsorption capacity under these acidic conditions [239]. These electrostatic interactions normally give poor selectivity in the adsorption step, and on increasing the ionic strength of the solution, the adsorption capacity of the targeted adsorbent is reduced [205]. 

In terms of the time-dependent adsorption, the kinetics of the adsorption process plays a significant role. The kinetics depends on different diffusional processes, including diffusion of the adsorbate from the bulk solution to the adsorbent–solution boundary, to the adsorbent surface, within the porous adsorbent and to the actual adsorption step. In most studies, the solution phase is agitated and this eliminates diffusional limitations within the bulk solution. Under these conditions, the intraparticle diffusion within the porous adsorbent tends to become the rate-determining step [198]. There is normally an initial rapid increase in the adsorption as the easily accessible adsorption sites are first reached and this is then followed by a more gradual increase until equilibrium adsorption is attained. The time required to achieve equilibrium can vary from about 200 min [198] to 58 h [139] for MB, and 500 min [240] to 20 h for MO [241], clearly highlighting the significant role of the diffusional processes within the adsorbents and the difficulty in comparing the different GO/CS adsorbents. Indeed, a direct comparison between the large number of GO/CS adsorbents employed, Table 4, Table 5 and Table 6, is very challenging given the significant variations in the properties of the chitosan used, including its DD levels, MW, porosity, particle size, the nature of the crosslinking agents employed during synthesis (Section 3) and the ratio of chitosan to GO and the presence of additional materials. 

### 5.2. Regeneration Strategies

The successful regeneration of the GO/CS-based adsorbents is an important consideration in the final applications of these materials. Efficient regeneration, without significant loss in the adsorption capacity, is not only necessary in terms of operating costs, but is also essential in the recovery of the adsorbates and the prevention of secondary waste. The desorbing agents employed in the regeneration of the GO/CS-based adsorbents are normally acids [229], bases [134,229], chelating agents, such as EDTA [213], or organic solvents, such as ethanol [166,198], methanol [176] or acetone [92]. The selection of the desorbing agent depends on the nature of the adsorbate. For example, NaOH is a good choice for the desorption of anionic dyes and various anions, whereas acidic eluents are effective in the removal of cationic dyes and cations, and organic solvents are suitable for the desorption of organic molecules. When an acidic eluent, such as HCl, is used, the amino groups in chitosan become protonated to give NH_3_^+^ and the –COO^–^ groups in GO become protonated to give –COOH. This favours the desorption of cationic species that were previously electrostatically bound to the –COO^–^ groups on GO. On the other hand, anionic species bound to the –NH_3_^+^ groups can be desorbed with an increase in pH as the –NH_3_^+^ is converted to –NH_2_ and the now negatively generated –COO^–^ groups will favour desorption of the anions. Indeed, solutions of NaOH have been employed to desorb anionic species such as chromate [180] and methyl orange [242], while HCl solutions have been employed to desorb divalent cations such as Pb(II) [175] and Cd(II) [243]. Organic solvents such as methanol have been employed in the desorption of ciprofloxacin [176] and acetone has been used to desorb methyl violet and Alizarin yellow R [92].

While these strategies can be employed in the regeneration of the GO/CS based adsorbents, the adsorption efficiency of the regenerated adsorbents tend to decrease with each regeneration step. For example, Zhao et al. [244] observed a gradual loss in the adsorption capacity and removal of phenol and p-nitrophenol on regeneration of the GO/CS-based adsorbent with NaOH. Furthermore, concentrated acids and bases are not always the best options in the regeneration of the GO/CS adsorbents. Magnetic particles, such as Fe_3_O_4_, tend to corrode and dissolve in acidic media. Therefore, EDTA has been employed in the regeneration step to protect magnetic particles [213]. 

However, it is well known that chitosan undergoes hydrolysis reactions in the presence of acids [96]. These hydrolysis reactions, which attack the polymer chains, give rise to a lowering in the MW and a reduction in the mechanical strength of chitosan. This makes the regeneration of GO/CS more complex and challenging, as the structure of the GO/CS will alter with repetitive regeneration steps. Furthermore, these changes in the overall structure of chitosan are likely to give rise to some restacking of the GO sheets, reducing the adsorption capacity. 

## 6. Conclusions

It is clearly evident from the increasing number of publications that focus on combining graphene and chitosan that these materials are emerging as interesting candidates for environmental applications. Both chitosan and graphene have good adsorption capacity. The addition of graphene to chitosan enhances its mechanical properties, while the chitosan hydrogel serves as an immobilization matrix for graphene. Fortunately, it is GO that has the best potential in the formulation of adsorbents, removing the need to use graphene in its pure form, which had been plagued with manufacturing issues. Other components, varying from supramolecular agents, magnetic nanoparticles and chelating agents, can also be added to the GO/CS hydrogel, to further enhance its properties.

However, this research is still in its early stages of development and a number of challenges exist and must be overcome before these materials can be employed as adsorbents in real applications. One of the more significant aspects from a health and well-being consideration is an evaluation of the toxic properties of GO. As increasing amounts of GO are used, it will eventually make its way into the aquatic environment, aided by its polar groups. There is increasing evidence to suggest that the build-up of GO within the aquatic environment may lead to bioaccumulation. Therefore, studies aimed at measuring the release and leaching of GO from the chitosan-based hydrogels are important. While chitosan can be employed as an immobilization matrix, it may be necessary to further modify the physiochemical properties of the chitosan hydrogel to trap more effectively the GO sheets. 

Another aspect that requires consideration is the final costs associated with the fabrication of the adsorbents and its potential for regeneration. The costs associated with the production of GO and the GO/CS adsorbent must be competitive in terms of the competing technologies and especially the competing activated carbon, which is currently the preferred adsorbent material. Ideally, adsorbents should be readily regenerated with little or no additional costs. With GO and the GO/CS composites, the adsorption capacity tends to decrease with each adsorption–regeneration cycle. Consequently, new regeneration strategies that will give rise to more cost-effective and efficient regeneration are needed. Furthermore, more studies should be focussed on the recovery of the adsorbate and especially the recovery of valuable metals that have accumulated within the composite. The recovery of valuable metals for re-use could be used to offset some of the adsorbent fabrication and recovery costs and contribute to the development of the GO/CS adsorbents as a sustainable technology. 

Selectivity in the adsorption process is another challenge as real water samples contain various ions, such as sodium, calcium and chlorides, that will consume the adsorption sites and thus reduce the removal capacity of the targeted pollutant or pollutants. Indeed, in many studies, an increase in the ionic strength gives rise to a reduction in the adsorption capacity of the targeted contaminant. Supramolecular chemistry, which not only includes cyclodextrins, but calixarenes and pillararenes, and imprinting technologies, has the potential to play a greater role in addressing these selectivity issues in the adsorption step. 

Magnetic GO/CS composites have the capacity to be removed following adsorption. Nevertheless, these magnetic materials must be sufficiently anchored within the GO/CS composites to prevent their leaching or dissolution and escape into the aquatic environment. Consequently, more studies are required aimed at monitoring the release of the magnetic/iron particles, Fe^2+^ and/or Fe^3+^, that could in turn lead to the development of sludge (insoluble iron hydroxide precipitates). Finally, batch experiments are normally employed with very few studies utilizing other techniques, such as fixed bed columns, that may be more applicable in real wastewater treatment applications. 

In conclusion, while these GO/CS adsorbents require further research and development, these materials have a promising future as adsorbent materials. Besides, new 2D materials are continuously being developed and these could be relatively easily combined with GO/CS to generate a new family of high performing and cost-effective adsorbents. 

## Figures and Tables

**Table 1:** Summary of some CD-modified GO adsorbents and their adsorption capacity.

Adsorbent	Adsorbate	Adsorption q_m_ (mg g^−1^)	Ref.
β-CD/GO	Bisphenol A	373.4	[72]
β-CD/GO	Methyl blue	580.4	[73]
	Methyl orange	328.2	
	Basic fuchsin	425.8	
β-CD/GO	Cd(II)	196.0	[74]
β-CD/GO	p-Nitrophenol	117.28	[75]
β-CD/poly(acrylic acid)/GO)	Methylene blue	247.99	[76]
	Safranine T	175.49	
β-CD/poly (L-glutamic acid) magnetic/GO	17β-estradiol	298.9	[77]

**Table 2:** Summary of the advantages and disadvantages of the synthetic processes.

Synthetic Step	Advantages/Disadvantages
Sol gel	Simple, other reagents can be easily added/mixing of GO and chitosan gives rise to an increase in solution viscosity, which can give rise to inhomogeneity in the final GO/CS hydrogel.
Hydrothermal	No need for crosslinking agents/some cost considerations with the relatively high temperatures in the vicinity of 120 °C.
Crosslinking Agents	Increase in mechanical properties/reduces the number of chelating sites that are required to bind and trap the pollutants and can be toxic.
Functionalised GO	Modifiable oxygenated functional groups of GO are ideal for functionalised, rich chemistry, can be used to crosslink single graphene sheets/synthesis can be time consuming.
Freeze drying	Scaffolds with defined pore size, highly suited to enhanced adsorption/freeze drying can be slow.

**Table 4:** Summary of some magnetic GO/CS adsorbents. The GO/CS is employed as a solid adsorbent in contact with the targeted pollutant dissolved in the solution phase.

Adsorbent	Adsorbate	Adsorption Conditions	Adsorption q_m_ (mg g^−1^)	Ref.
MGO/CS	Rifampicin	Batch mode, 55 °C, 200 rpm, 10 mg adsorbent, 20 mg/L adsorbate	102.11	[166]
MGO/CS	As(III)	Batch, 25 °C, 250 rpm, 10 mg/L adsorbate, V = 100 mL	45	[178]
MGO/CS	Methylene blue Eriochrome black T	Batch, 26 °C, 130 rpm, 50 mg adsorbent, 100 mg/L adsorbate, V = 50 mL	289 292	[183]
MGO/CS	Cr(VI)	Batch, 21 °C, 500 mg adsorbent, 40 mg/L adsorbate, V = 10 mL	100.51	[184]
GO/CS/ZnFe_2_O_4_	Basic fuchsin	Batch, 25 °C, 50 mg adsorbent, 50 mg/L adsorbate, V = 25 mL	335.57	[185]
Methacrylic acid functionalized-MGO/CS	Methylene blue	Batch, 25 °C, 120 rpm, 10 mg adsorbent, 100 mg/L adsorbate, V = 20 mL	2478	[162]
MGO/CS/ Lignosulfonate	Methylene blue	Batch, 30 °C, 160 rpm, 10 mg adsorbent, V = 20 mL	253.53	[186]
MGO/CS/ Ethylenediamine	Cu(II)	Batch, 25 °C, 300 rpm, 10 mg adsorbent, 100 mg/L adsorbate, V = 50 mL	217.4	[187]
MGO/CS/SiO_2_	Dopamine Clenbuterol Orciprenaline Methylene blue Crystal violet	Batch, 20 °C, 180 rpm, 10 mg adsorbent, V = 100 mL	127.34 109.56 150.21 300.42 347.35	[171]
MGO/CS/SiO_2_	methyl violet	Batch, 52 °C, 150 rpm, 10 mg adsorbent, 10 mg/L adsorbate, V = 5 mL	243.8	[188]
MGO/CS/SiO_2_/ ionic liquid	Morphine Codeine Ephedrine Amphetamine Benzoylecgonine	Batch, 25 °C, 150 rpm, 15 mg adsorbent, 10 mg/L adsorbate, V = 5 mL	7.2 8.4 9.2 5.8 11.2	[189]
3D-MGO/CS	Disperse blue 367	Batch, 25 °C, 150 rpm, 150 mg adsorbent, 60 mg/L adsorbate	298.27	[190]
β-CD-MGO/CS	Bisphenol A Bisphenol F	Batch, 30 °C, 200 rpm, 20 mg adsorbent, 20 mg/L adsorbate, V = 50 mL	326.8 328.3	[191]
β-CD–MGO/CS	Hydroquinone	Batch mode, 180 rpm, 100 mg adsorbent, V = 100 mL	148	[182]
β-CD–MGO/CS	Cr(VI)	Batch, 180 rpm, 100 mg adsorbent, 50 mg/L adsorbate, V = 100 mL	67.66	[181]
β-CD–MGO/CS	Cr(VI)	Batch, 150 rpm, 100 mg adsorbent, 100 mg/L adsorbate, V = 100 mL	120	[192]
β-CD–MGO/CS	Malachite green	Batch, 25 °C, 150 rpm, 5 mg adsorbent, V = 20 mL	740.74	[60]
β-CD–MGO/CS	p-Phenylene-diamine	Batch, 45 °C, 5 mg adsorbent, 100 mg/L adsorbate, V = 20 mL	1102.58	[193]
β-CD–MGO/CS	Methylene blue	Batch, 25 °C, 180 rpm, 10 mg adsorbent, V = 25 mL	43.34	[177]

**Table 6:** Adsorption capacity of blended chitosan biopolymers combined with GO, where the solid adsorbent is added to the pollutant-containing solution phase.

Blended Polymer	Adsorbent/Adsorption Experiment	Adsorbate	Adsorption q_m_ (mg g^−1^)	Ref.
PVA (poly(vinyl alcohol))	GO/CS/PVA Batch, 30 °C, 160 rpm, 20 mg adsorbent, V = 40 mL	Cd(II) Ni(II)	172.11 70.37	[230]
PVA	GO/CS/PVA Batch, 140 rpm, 6 g/L adsorbent	Congo red dye	12.38	[226]
PVA	GO/CS/PVA Batch, 22.16 mg/L adsorbate, 0.5 g/L adsorbent	Sr(II)	17.48	[231]
PVA	GO/CS/PVA Batch, 30 °C, 150 rpm, 50 mg adsorbent, V = 100 mL	Cu(II)	162	[232]
PAA (polyacrylic acid)	GO/CS/PAA Batch, 25 °C, 0.2 g adsorbent, V = 150 mL	Rhodamine 6G Methyl violet Methyl orange	224.6 169.2 195.6	[227]
PAA	GO/CS/PAA/Fe_3_O_4_ Batch, 25 °C, 300 rpm, 10 mg adsorbent, V = 50 mL	Cu(II)	217.4	[187]
PLA (polylactic acid)	GO/CS/PLA Batch, 110 rpm, 25–45 °C, 30 mg adsorbent, V = 30 mL	Crystal violet	45	[201]
CMC (carboxymethyl cellulose)	GO/CMC/CS Batch, 25 °C, 200 rpm, 5 mg adsorbent, V = 20 mL	Sulfameth- oxazole Sulfapyridine	312.2 161.8	[233]
CMC	GO/CS/CMC Batch, 25 °C, 0.4–0.6 g/L adsorbent	MB MO	655.98 404.52	[229]
C Cellulose)	GO/CS/C Batch, 30 °C, 200 rpm, 5 mg adsorbent, V = 8 mL	Cu(II)	22.40	[228]
SA (sodium alginate)	GO/SA/CS/FeO Batch, 30 °C, 50 mg adsorbent	Cu(II) Cd(II) Pb(II)	55.96 86.28 189.04	[169]

## Data Availability

Not applicable.
